# Mitral Transcatheter edge-to-edge repair rivals surgery for survival despite less complete correction: a systematic review and metanalysis of randomized and propensity score matching studies

**DOI:** 10.1093/ehjopen/oeaf135

**Published:** 2025-10-25

**Authors:** Gianluca Di Pietro, Riccardo Improta, Antonio Lattanzio, Alessandro Roscioli, Lucia Ilaria Birtolo, Marco Tocci, Riccardo Colantonio, Gennaro Sardella, Silvio Fedele, Natalia Pavone, Wael Saade, Fabio Miraldi, Massimo Mancone

**Affiliations:** Department of Clinical, Internal, Anesthesiological and Cardiovascular Sciences, Umberto I Hospital, La Sapienza University of Rome, Viale del Policlinico 155, Rome 00161, Italy; Department of Clinical, Internal, Anesthesiological and Cardiovascular Sciences, Umberto I Hospital, La Sapienza University of Rome, Viale del Policlinico 155, Rome 00161, Italy; Department of Clinical, Internal, Anesthesiological and Cardiovascular Sciences, Umberto I Hospital, La Sapienza University of Rome, Viale del Policlinico 155, Rome 00161, Italy; Department of Clinical, Internal, Anesthesiological and Cardiovascular Sciences, Umberto I Hospital, La Sapienza University of Rome, Viale del Policlinico 155, Rome 00161, Italy; Department of Clinical, Internal, Anesthesiological and Cardiovascular Sciences, Umberto I Hospital, La Sapienza University of Rome, Viale del Policlinico 155, Rome 00161, Italy; Department of Clinical, Internal, Anesthesiological and Cardiovascular Sciences, Umberto I Hospital, La Sapienza University of Rome, Viale del Policlinico 155, Rome 00161, Italy; Department of Clinical, Internal, Anesthesiological and Cardiovascular Sciences, Umberto I Hospital, La Sapienza University of Rome, Viale del Policlinico 155, Rome 00161, Italy; Department of Clinical, Internal, Anesthesiological and Cardiovascular Sciences, Umberto I Hospital, La Sapienza University of Rome, Viale del Policlinico 155, Rome 00161, Italy; Interventional Cardiology Unit, Sandro Pertini Hospital, Via dei Monti TIbrutini 385, Rome 00158, Italy; Department of Cardiovascular Medicine, Fondazione Policlinico Universitario A. Gemelli IRCCS, Largo Agostino Gemelli, Rome 00168, Italy; Department of Clinical, Internal, Anesthesiological and Cardiovascular Sciences, Umberto I Hospital, La Sapienza University of Rome, Viale del Policlinico 155, Rome 00161, Italy; Department of Clinical, Internal, Anesthesiological and Cardiovascular Sciences, Umberto I Hospital, La Sapienza University of Rome, Viale del Policlinico 155, Rome 00161, Italy; Department of Clinical, Internal, Anesthesiological and Cardiovascular Sciences, Umberto I Hospital, La Sapienza University of Rome, Viale del Policlinico 155, Rome 00161, Italy

**Keywords:** mitral regurgitation, Transcatheter edge-to-edge, Surgery, Comparison, Mid-term outcomes

## Abstract

**Aims:**

To compare outcomes of patients with severe mitral regurgitation (MR) after m-TEER and surgery.

**Methods and results:**

PubMed, Scopus, and Google Scholar databases were searched for randomized controlled trials and propensity score matching studies comparing mid-term outcomes of m-TEER vs. surgical valve repair. All-cause of death, rehospitalization for heart failure, mitral reintervention, NYHA class at clinical follow-up and grade ≥ 3 at echocardiographic follow-up were the outcomes of interest. Additional sensitivity analyses were performed to account for heterogeneity. Nine studies (2 RCT and 7 propensity score matching studies) with a total of 23 825 patients (m-TEER group = 11 970; surgery group = 11 855) were included. Surgery and m-TEER were associated with comparable rates of all-cause mortality at a median follow-up of 18 months (RR 1.02, 95%CI 0.77–1.37, *P*-value 0.87). Surgical repair was associated with a reduced risk of rehospitalization for heart failure (RR 1.70, 95%CI 1.47–1.98, *P* value < 0.01) and mitral reintervention (RR 3.27, 95%CI 2.49–4.30, *P* value < 0.01), due to a reduced at least moderate residual MR (RR 6.35, 95%CI 1.43–28.22, *P* value 0.02).

**Conclusion:**

In patients with severe MR, m-TEER resulted in comparable outcomes for all-cause deaths compared to surgery, although the latter was associated with reductions in heart failure rehospitalization, reintervention and MR residual rates at a median 18-month follow-up.

## Graphical Abstract

**Graphical Abstract:**
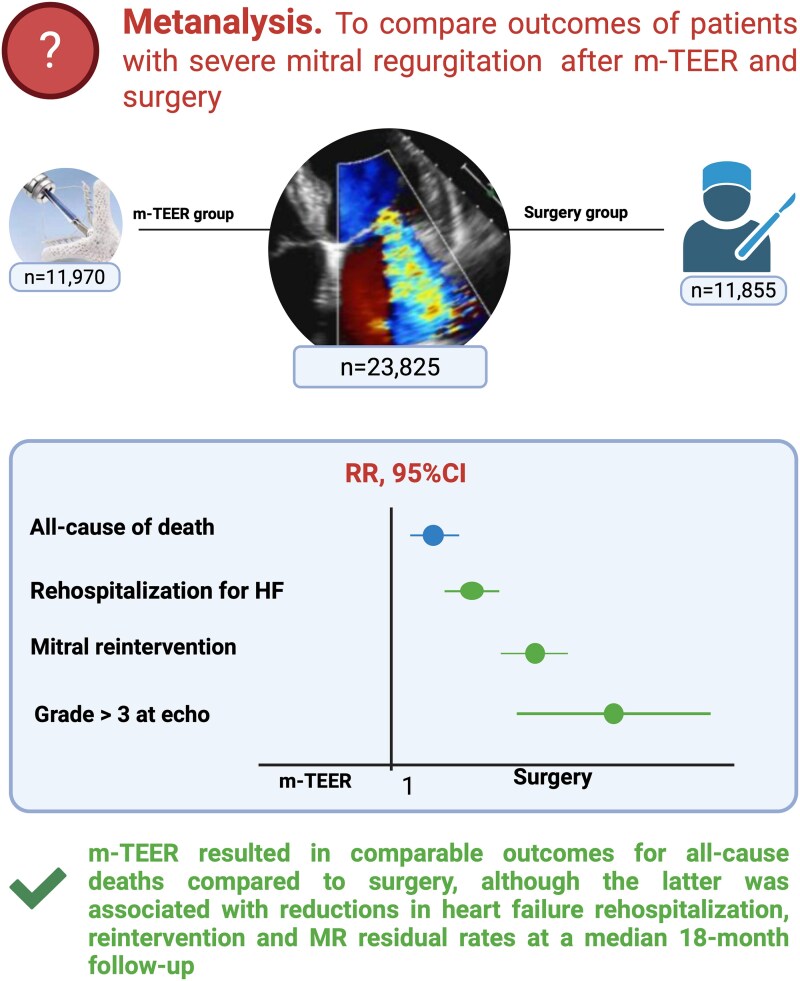

## Introduction

Despite mitral regurgitation (MR) being the most common heart valve disease worldwide with a growing burden^1^ and worsening outcomes,^2^ the 2019 EURObservational VHD II Survey showed that too many patients with severe MR remain undertreated.^3^ Surgical treatment represents the preferred approach, especially in symptomatic patients with severe MR and acceptable operative risk.^4^ Transcatheter edge-to-edge repair (TEER) has revolutionized the management of MR offering a minimally invasive alternative. Historical data from the EVEREST II study showed comparable long-term mortality rates for TEER as compared with the surgical approach.^5^ Meanwhile, commercial devices and techniques have progressively improved so much that new international guidelines reserved m-TEER for both degenerative and functional severe MR with a IIa recommendation.^6^

This upgrade recommendation is mainly based on registry studies without an extensive head-to-head comparison evidence.^7,8^ Unlike transcatheter aortic valve implantation (TAVI), where trials systematically compared percutaneous procedure to surgery from the outset, studies on transcatheter mitral repair have rarely pitted it against surgery, reflecting these divergent patient profiles (including age or surgical risk). For these reasons, the High and Intermediate Risk Degenerative Mitral Regurgitation Treatment (HiRiDe) trial was prematurely terminated due to severe enrolling difficulties.^9^

Given this lack of knowledge, this metanalysis of randomized controlled trials (RCTs) and propensity match score (PMS) studies aimed to compare outcomes following m-TEER or surgery.

## Method

The present analysis was conducted in accordance with Preferred Reporting Items for Systematic Reviews and Meta-analyses (PRISMA) guidelines^10^ and was preregistered in the international prospective register of systematic reviews (PROSPERO CRD420251027144). The data that support the findings of this study are available from the corresponding author upon reasonable request. Approval from institutional review board for this study was waived because of the lack of individual patient information. Patient written consent for the publication of the study was not received because of the lack of individual patient information.

### Search study

Comprehensive searches to identify all RCTs and PSM studies that assessed long-term outcomes of patients underwent m-TEER and surgery (repair and/or replacement) was performed. Searches were run on 20 March 2025 in the following databases: PubMed Central, Ovid EMBASE, and Google scholar. The search strategy included the following terms: ‘mitral regurgitation’, ‘primary mitral regurgitation’, ‘degenerative mitral regurgitation’, ‘functional mitral regurgitation’, ‘secondary mitral regurgitation’, ‘randomized controlled trials’, ‘propensity score match studies’, ‘RCTs’, ‘PSM’.

### Study selection and data extraction

**Figure 1:**
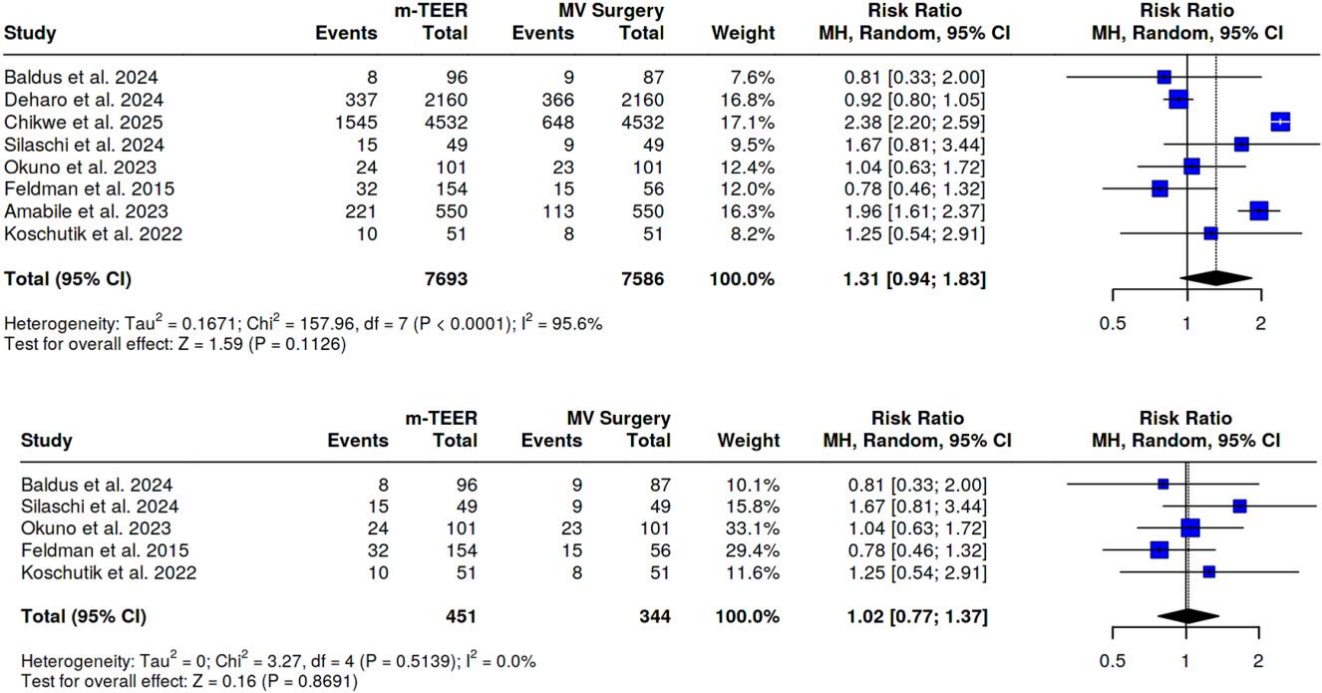
All cause of death. (*A*) Primary analysis; (*B*) sensitivity analysis.

Database searches, screening, and exclusion of duplicated results were performed by two physicians (A.L., A.R.). Two investigators (G.D.P., R.I.) screened the searched database for inclusion and performed data extraction independently. Disagreements were resolved by a third author, who also checked the extracted data for accuracy (A.C.). Studies were considered for inclusion in the quantitative analysis if they were RCTs or PSM studies written in English which compared mid-term outcomes of m-TEER vs. surgical valve repair or replacement. For studies with overlapping sample, the publication with the largest cohort was selected. Studies that did not report the overall sample size of matched cohorts and those with short-term outcomes were excluded. See Supplementary material online, *Appendix S1*  *and S2*. Additionally, animal studies, case reports, conference presentations, editorials, reviews, and expert opinions were also excluded. Full text for the selected studies was reviewed for a second round of eligibility screening. Reference lists of articles were also searched to identify other relevant studies. The PRISMA flow diagram of the study selection process is shown in *Figure 1*. Data on relevant baseline variables for each of the included studies were extracted for the m-TEER and surgery groups. The quality of the included papers was assessed using the ROBINS-I tool^11^ for PSM studies and ROB2 tool^12^ for RCTs. Publication bias was assessed by visual inspection of funnel plots and Egger Test.

### Outcomes

Outcomes of interests were the following: all-cause of death (defined as the mortality rate for a population across all causes); rehospitalization for heart failure (HF); mitral reintervention; NYHA class clinical follow-up; at least grade 3+ at echocardiographic follow-up.

### Statistical analysis

Continuous variables are reported as mean (standard deviation) or median (first [Q1] and third quartile [Q3]). Categorical variables are expressed as *n* (%). Statistical pooling for incidence estimates was performed using a random effect model with generic inverse variance weighting, computing risk estimates with 95% confidence intervals (CIs), using RevMan 5.2 (The Cochrane Collaboration, The Nordic Cochrane Centre, Copenhagen, Denmark). The selection of a random-effects model was driven by the need to aptly address the inherent heterogeneity observed within the pool of included PSM studies. Hypothesis testing for superiority was conducted at a two-tailed significance level of 0.05. We used the I2 statistic to assess heterogeneity. Low heterogeneity was defined as 0–25%; moderate heterogeneity was defined as 25–50%; and substantial heterogeneity was defined as >50%. An additional sensitivity analysis was performed in the presence of significant heterogeneity.

## Results

Nine studies (2 RCTs and 7 PSM studies) 5, 13, 14, 15, 16, 17, 18, 19, 20 with a total of 23 825 patients (11 970 m-TEER and 11 855 surgery) were included in the quantitative analysis. The characteristics of the included studies are shown in *Table 1*. A complete reference list of included studies is provided in Supplementary material online, Appendix List 2. Baseline clinical characteristics of included patients are summarized in *Table 2*. In brief, the median age was 74 (IQR 71–77) years for m-TEER patients and 72 (IQR 70–76) years for surgery patients. In the surgical arm, mitral valve replacement was performed in 1.6% (IQR 0–26.7). Patients undergoing m-TEER were characterized by a higher burden of cardiovascular risk factors compared to controls: 59% of patients were dyslipidaemia and 30% diabetic in the m-TEER cohort, compared with a prevalence of dyslipidaemia and diabetes of 49% and 19% in the surgery cohort. Patients scheduled for m-TEER were more likely to have previously undergone percutaneous coronary intervention (33%, IQR 16–47) compared to surgery (14%, IQR 5–20). The EUROSCORE II perioperative risk score was similar between the cohorts (3.9%, IQR 2–7% vs. 3.9% IQR 2–5). A descriptive visualization of baseline distributions using violin plots, generated from study-level medians and IQRs, is provided in Supplementary material online, *Figure S1*.

**Table 1:** Characteristics of included studies

Authors	Design of the study	Year of publication	Enrolment Years	Follow-up (months)	Sample Size (n)	m-TEER (n)	Surgery (n)	Surgical replacement (%)	MR aetiology
Deharo et al.^13^	PSM	2024	2012–2022	12	4320	2160	2160	25.4	Degenerative: 40% in MVS, 40.6% in m-TEER
Baldus et al.^14^	RCT	2024	2015–2022	12	208	104	104	28	Functional: 100%
Okuno et al.^15^	PSM	2023	2012–2018	24	202	101	101	0	Functional: 100%
Majmundar et al.^16^	PSM	2024	2016–2019	12	8521	4269	4252	0	Degenerative:50%
Koschutnik et al.^17^	PSM	2022	2017–2020	24	102	51	51	33	Degenerative: 84% in MVS, 39% in m-TEER
Feldman et al.^5^	RCT	2015	2005–2008	60	210	154	56	14	Degenerative: 77.5% in MVS, 73% in m-TEER
Silaschi et al.^18^	PSM	2024	2010–2021	12	98	49	49	1.6	Degenerative: 81.6% in MVS and m-TEER
Amabile et al.^19^	PSM	2023	2015–2020	36	1100	550	550	0	Degenerative: NA
Chikwe et al.^20^	PSM	2025	2012–2019	34	9064	4532	4532	0	Degenerative: 100%

Abbreviations: MR, mitral regurgitation; PSM, propensity score matching; RCT, randomized controlled trial; TEER, transcatheter edge-to-edge repair.

**Table 2:** Baseline characteristics of patients

	m-TEER (*n* = 11 970)	Surgery (*n* = 11 855)
Age, years old (IQR)	74 [71–77]	72 [70–76]
Men, % (IQR)	57 [43–63]	57 [49–66]
Hypertension, % (IQR)	84 [83–98]	83 [82–98]
Diabetes, % (IQR)	30 [13–73]	19 [11–72]
Dyslipidaemia, % (IQR)	59 [40–72]	49 [38–60]
BMI, kg/m^2^ (IQR)	26 [25–28]	28 [26–28]
Heart failure,% (IQR)	81 [77–95]	78 [77–90]
NYHA classes > III, % (IQR)	63 [54–84]	67 [47–83]
CAD, % (IQR)	47 [33–49]	43 [23–49]
Previous MI, %(IQR)	22[11–33]	13 [7–28]
Previous PCI, % (IQR)	33 [16–47]	14 [5–20]
Previous CABG, % (IQR)	16 [5–50]	16 [3–65]
AFib/Flutter, % (IQR)	50 [34–69]	44 [36–60]
Stroke, %(IQR)	6 [5–8]	7 [4–10]
Previous CIED, %(IQR)	16 [12–22]	10 [2–18]
COPD, % (IQR)	22 [16–30]	14 [12–29]
CKD, % (IQR)	36 [19–60]	35 [17–58]
EUROSCORE II, %(IQR)	3,9 [2–7]	3,9 [2–5]
Surgical mitral replacement, %(IQR)	–	1,6 [0–26,7]

Abbreviations: AFib, atrial fibrillation; BMI, body mass index; CABG, coronary artery by-pass grafting; CAD, coronary artery disease; CIED, cardiovascular implantable electronic device; COPD, chronic obstructive pulmonary disease; CKD, chronic kidney disease; IQR, interquartile range; MI, myocardial infarction; PCI, percutaneous coronary intervention.

The risk of bias assessment for the included studies is shown in Supplementary material online, *Figure S1*. Funnel plot for visual inspection of publication bias all-cause of death is shown in Supplementary material online, *Figure S2*.

### All-cause of death

Eight studies, encompassing 15 286 patients (7693 m-TEER and 7586 surgery) assessed the rates of all-cause of death at a median follow up of 18 months (IQR 12–34) among patients underwent m-TEER compared with controls. m-TEER patients showed a comparable risk of all-cause of death as compared with the surgery cohort (RR 1.31, 95%CI 0.94–1.83, *P-*value 0.11). The Egger’s test did not support the presence of asymmetry (intercept −2.33, *P-*value 0.37). However, significant heterogeneity was observed (*I*^2^ = 96%). The results were not affected after removing outlier studies (RR 1.02, 95%CI 0.77–1.37, *P*-value 0.87, *I*^2^ = 0%). See *Figures 1A* and *B*.

### Rehospitalization for heart failure

**Figure 2:**
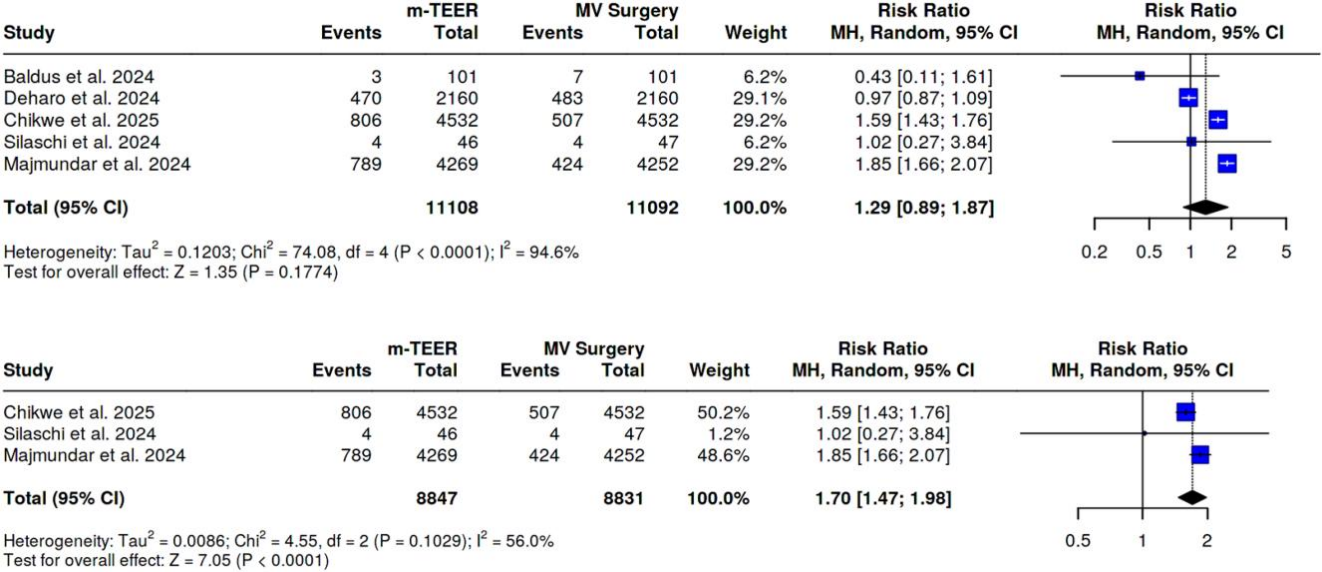
Rehospitalization for HF. (*A*) primary analysis; (*B*) sensitivity analysis.

Pooled estimates from five studies showed that m-TEER patients have similar risk of rehospitalization for HF as compared with controls (RR 1.29, 95%CI 0.89–1.87, *P*-value 0.18) with significant heterogeneity (*I*^2^ = 95%). The Egger’s test did not support the presence of funnel plot asymmetry (intercept −1.71, *P-*value 0.87). However, after removing outlier studies, m-TEER patients experienced an increased risk of rehospitalization for HF (RR 1.70, 95%CI 1.47–1.98, *P-*value < 0.01) without a significant heterogeneity (*I*^2^ = 56%, *P-*value 0.10). See *Figures 2A* and *B*.

### Mitral reintervention

**Figure 3:**
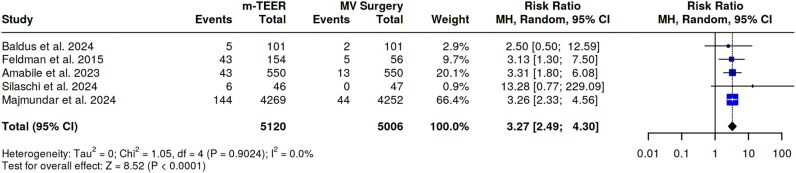
Mitral reintervention.

Overall, five studies including 10 119 patients (m-TEER group: 5120 patients and surgery group = 5006 patients) assessed the rates of mitral reintervention at a median follow-up of 18 months. Patients scheduled for m-TEER showed an increased risk of mitral reintervention than those who received surgery (RR 3.27, 95%CI 2.49–4.30, *P*-value < 0.01) without significant heterogeneity (*I*^2^ = 0%). The Egger’s test did not support the presence of funnel plot asymmetry (intercept −0.75, *P*-value 0.685). See *Figure 3*.

In an exploratory subgroup analysis restricted to the three studies in which the surgical cohort consisted exclusively of mitral valve repair, m-TEER was associated with a higher risk of mitral reintervention compared to mitral valve repair (RR 3.32, 95%CI 2.48–4.44, *P*-value < 0.0001), with no significant heterogeneity (*I*² = 0%). See Supplementary material online, *Figure S4*. These findings are consistent with the main pooled analysis, suggesting that the small proportion of replacements in the overall surgical group may not significantly affect the observed outcome.

### Grade ≥ 3 at echocardiographic follow-up

Pooled estimates from three studies showed m-TTER was associated with an increased risk of having at least moderate regurgitation at echocardiographic follow-up compared with controls (RR 6.35, 95%CI 1.43–28.22, *P*-value 0.02, *I*^2^ = 0%). See Supplementary material online, *Figure S5A*. A visual representation of the pooled proportions for each group is provided in Supplementary material online, *Figure S5B*, illustrating the magnitude of this difference.

### NYHA class ≥ 3 at clinical follow-up

Pooled estimates from three studies showed that patients scheduled for m-TEER had a comparable risk of experiencing a moderate functional impairment (according to NYHA class) as compared to controls (RR 1.07, 95%CI 0.37–3.08, *P*-value = 0.89) with non-significant heterogeneity (*I*^2^ = 64%, *P*-value 0.06). See Supplementary material online, *Figure S6*.

### Beyond the EVEREST II Trial

Published in 2011, the EVEREST II trial was considered a landmark study comparing MitraClip to conventional surgical treatment. While it demonstrated the feasibility and safety of MitraClip, the study is now considered outdated due to advances in device technology. Its restrictive inclusion criteria and early context limit its relevance to modern practice. All-cause mortality rates (RR 1.40, 95%CI 0.90–2.17, *P*-value 0.13 and RR 1.17, 95%CI 0.83–1.65, *P*-value 0.36) were unaffected after the EVEREST trial was removed. See Supplementary material online, *Figures S7A* and *B*. Similarly, m-TEER was associated with higher rates of mitral reintervention compared with the surgical approach, even after removing the EVEREST trial (RR 3.29, 95%CI 2.47–4.38, *P*-value < 0.01). See Supplementary material online, *Figures S8*.

## Discussion

This systematic review and metanalysis, including randomized trials and propensity score studies, compared m-TEER outcomes with conventional surgical treatment in patients with severe mitral valve regurgitation.

The main results can be summarized as follows (*Table 3*, *Graphical abstract*):

(1) All-cause of mortality was comparable between the two strategies at a median follow-up of 18 months.
(2) Patients underwent m-TEER experienced a significant risk of HF readmissions, mitral valve reintervention, and at least moderate postoperative recurrent mitral valve regurgitation as compared to those undergoing surgical repair.

**Table 3:** Summary of results

	RR [95%CI]
All-cause of mortality	1.02 [0.77–1.37]
Rehospitalization for heart failure	1.70 [1.47–1.98]
Mitral reintervention	3.27 [2.49–4.30]
Grade ≥ 3 at echocardiographic follow-up	6.35 [1.43–28.22]
NYHA Class ≥ 3 at clinical follow-up	1.07 [0.37–3.08]

Correction of MR consistently improves clinical outcomes compared with untreated MR in a variety of clinical settings, highlighting the adverse natural history of uncorrected disease and the prognostic benefit of mitral valve repair. Indeed, previous studies have clearly shown that surgical or transcatheter edge-to-edge repair reduced all-cause of mortality and rehospitalization rates both in degenerative MR^5^ and in secondary MR due to HF with reduced ejection fraction (HFrEF).^21,22^ Despite its clinical benefits, percutaneous mitral valve repair has not been widely supported over surgery in statistically powered randomized trials. This highlights a clear gap with TAVR, which is established over surgery. The highly heterogeneous patient populations or pathophysiological mechanisms and the less straightforward outcomes to assess are the challenges in conducting RCTs for MR.

In addition, most of the studies available in the literature often did not distinguish between the various mechanisms of MR. Although phenotyping of patients is crucial, this lack of differentiation occurred because sample sizes were often insufficient to perform robust sub-analyses, the distinction between primary and secondary MR was often more nuanced, particularly in older studies, and many studies were designed to assess the overall efficacy of m-TEER vs. surgery in a broad MR population, with subgroup analyses being a secondary objective. These findings form the basis of our meta-analysis, which shows nuanced differences in the effectiveness of m-TEER and surgery.

Our results suggest that both m-TEER and surgery offer similar mid-term survival benefits, consistent with the MATTERHORN trial, which reported no significant difference in mortality in secondary MR at short-term follow-up. The TRAMI registry reported a 1-year mortality rate of approximately 20%, comparable to surgical outcomes in propensity-matched analyses, reinforcing our observations that neither approach confers a clear survival advantage.^8^

Beyond mortality, the issue of residual MR is directly related to these rehospitalization trends and is a critical differentiator in our analysis, with surgery showing a reduced long-term risk of moderate MR along with fewer reinterventions. This is likely due to its ability to achieve a more complete and durable correction of MR, reducing the downstream burden of volume overload that drives HF exacerbations. Indeed, during open-heart surgery, physiological restoration of mitral valve function is typically achieved through complex repair techniques, including scallop resection, leaflets sliding, and annuloplasty with prosthetic ring implantation; in cases where repair is not feasible, complete valve replacement remains the preferred alternative.

The greater durability observed with surgery could, at least in part, be influenced by the inclusion of valve replacement procedures, which inherently prevent recurrent MR. However, such procedures were uncommon across the studies included in this meta-analysis (median 1.6%, IQR 0–26.7%). An exploratory analysis restricted to repair-only cohorts yielded results consistent with the overall findings for mitral reintervention, suggesting that this factor is unlikely to have played a significant role.

The EVEREST II trial,^5^ the EuroSMR,^7^ and TRAMI^8^ Registries resulted in significantly less residual MR after surgery at longest follow-up, a factor that correlates with fewer hospitalizations. Similarly, Buzzati et al. reported a higher incidence of significant MR recurrence at 2 years with m-TEER compared to surgery, aligning with our observation of durability of surgery.^23^ The TVT Registry also reported a 12.15% reintervention rate at 5 years post-m-TEER, often due to recurrent MR, while the STS Registry showed rates of 2–5% with surgery.^24^

The forthcoming results of the MITRA-HR trial^25^ and the PRIMARY study^26^ will provide much-needed randomized evidence on the comparative effectiveness of M-TEER and surgical repair for treating severe MR in both high-risk and standard-risk patients. These investigations will help to determine whether the survival equivalence observed in our meta-analysis can be confirmed in prospectively enrolled populations by filling current gaps in trial data. They will also better quantify the balance between procedural durability and perioperative risk. The findings of these studies are likely to play a decisive role in refining patient selection, informing guideline recommendations and supporting heart team decision-making in the coming years.

Although the aim of this meta-analysis is to partially fill a gap in the evidence by directly comparing m-TEER and surgery, several limitations must be considered.

First, we included RCTs and real-world propensity score-matched observational studies. Although including PSM studies increases the generalizability of the findings by encompassing broader and more contemporary patient populations, it inevitably introduces variability related to differences in study design, data sources, and outcome ascertainment. Furthermore, PSM can only adjust for measured covariates and cannot account for unmeasured confounders such as frailty, anatomical complexity or subtle clinical features that may influence treatment allocation and outcomes. This limitation is particularly relevant given that m-TEER is often offered to patients for whom surgery is contraindicated, resulting in baseline differences that may persist despite matching.^27^ These factors should therefore be considered when interpreting the results of the present analysis.

Second, a small proportion of surgical patients underwent valve replacement, which may influence outcomes such as residual MR and reintervention. Third, the heterogeneity of MR aetiology poses a challenge, as the included studies often do not stratify outcomes by aetiology. This lack of stratification may obscure differential treatment effects, as primary and secondary MR have different pathophysiological mechanisms and responses to repair, potentially affecting secondary endpoints such as residual MR and rehospitalizations. Fourth, the median follow-up of 18 months may not be sufficient to capture the full durability benefit of surgery, as studies such as EVEREST II suggest that benefits become more pronounced beyond 5–10 years. Fifth, baseline risk differences between the cohorts may bias secondary outcomes despite propensity score adjustment. Sixth, variability in operator experience and procedural techniques between trials may introduce confounding. Finally, the lack of standardized definitions or reporting of residual MR severity and HF hospitalization criteria across studies introduces inconsistencies in outcome assessment. Future analyses stratifying by MR aetiology, extending follow-up and standardizing outcome definitions could address these issues and refine our findings.

## Conclusion

In conclusion, in patients with severe MR, m-TEER was not inferior to surgery in terms of all-cause mortality, although this observation period may be too short to detect a mortality benefit. Evidently, the surgical approach was associated with a reduction in HF rehospitalization, reintervention, and residual MR rates at a median follow-up of 18 months. Although m-TEER is a promising and rapidly evolving technique, its primary role remains in patients with high surgical risk or with a reduced life expectancy. Further studies in different patient cohorts are warranted to confirm our findings.

## Supplementary Material

oeaf135_Supplementary_Data

## Data Availability

The datasets generated and analysed during the current study are available from the corresponding author on reasonable request.

## References

[1] Nkomo VT, Gardin JM, Skelton TN, Gottdiener JS, Scott CG, Enriquez-Sarano M. Burden of valvular heart diseases: a population-based study. Lancet 2006;368:1005–1011. 16980116 10.1016/S0140-6736(06)69208-8

[2] Dziadzko V, Clavel MA, Dziadzko M, Medina-Inojosa JR, Michelena H, Maalouf J, Nkomo V, Thapa P, Enriquez-Sarano M. Outcome and undertreatment of mitral regurgitation: a community cohort study. Lancet 2018;391:960–969. 29536860 10.1016/S0140-6736(18)30473-2 PMC5907494

[3] Lung B, Delgado V, Rosenhek R, Price S, Prendergast B, Wendler O, De Bonis M, Tribouilloy C, Evangelista A, Bogachev-Prokophiev A, Apor A, Ince H, Laroche C, Popescu BA, Piérard L, Haude M, Hindricks G, Ruschitzka F, Windecker S, Bax JJ, Maggioni A, Vahanian A; EORP VHD II Investigators. Contemporary presentation and management of valvular heart disease: the EURObservational research programme valvular heart disease II survey. Circulation 2019;140:1156–1169. 31510787 10.1161/CIRCULATIONAHA.119.041080

[4] Vahanian A, Beyersdorf F, Praz F, Milojevic M, Baldus S, Bauersachs J, Capodanno D, Conradi L, De Bonis M, De Paulis R, Delgado V, Freemantle N, Gilard M, Haugaa KH, Jeppsson A, Jüni P, Pierard L, Prendergast BD, Sádaba JR, Tribouilloy C, Wojakowski W; ESC/EACTS Scientific Document Group. 2021 ESC/EACTS guidelines for the management of valvular heart disease. Eur Heart J 2022;43:561–632. Erratum in: Eur Heart J. 2022 Jun 1;43(21):2022. 34453165 10.1093/eurheartj/ehab395

[5] Feldman T, Kar S, Elmariah S, Smart SC, Trento A, Siegel RJ, Apruzzese P, Fail P, Rinaldi MJ, Smalling RW, Hermiller JB, Heimansohn D, Gray WA, Grayburn PA, Mack MJ, Lim DS, Ailawadi G, Herrmann HC, Acker MA, Silvestry FE, Foster E, Wang A, Glower DD, Mauri L; EVEREST II Investigators. Randomized comparison of percutaneous repair and surgery for mitral regurgitation: 5-year results of EVEREST II. J Am Coll Cardiol 2015;66:2844–2854. 26718672 10.1016/j.jacc.2015.10.018

[6] Otto CM, Nishimura RA, Bonow RO, Carabello BA, Erwin JP 3rd, Gentile F, Jneid H, Krieger EV, Mack M, McLeod C, O'Gara PT, Rigolin VH, Sundt TM 3rd, Thompson A, Toly C. 2020 ACC/AHA guideline for the management of patients with valvular heart disease: executive summary: a report of the American College of Cardiology/American Heart Association joint committee on clinical practice guidelines. Circulation 2021;143:932. 10.1161/CIR.0000000000000932 33332149

[7] Kalbacher D, Schäfer U, Bardeleben RS V, Eggebrecht H, Sievert H, Nickenig G, Butter C, May AE, Bekeredjian R, Ouarrak T, Kuck KH, Plicht B, Zahn R, Baldus S, Ince H, Schillinger W, Boekstegers P, Senges J, Lubos E. Long-term outcome, survival and predictors of mortality after MitraClip therapy: results from the German transcatheter mitral valve interventions (TRAMI) registry. Int J Cardiol 2019;277:35–41. 30153994 10.1016/j.ijcard.2018.08.023

[8] Stocker TJ, Stolz L, Karam N, Kalbacher D, Koell B, Trenkwalder T, Xhepa E, Adamo M, Spieker M, Horn P, Butter C, Weckbach LT, Novotny J, Melica B, Giannini C, von Bardeleben RS, Pfister R, Praz F, Lurz P, Rudolph V, Metra M, Hausleiter J; EuroSMR Investigators. Long-term outcomes after edge-to-edge repair of secondary mitral regurgitation: 5-year results from the EuroSMR registry. JACC Cardiovasc Interv 2024;17:2543–2554. 39537275 10.1016/j.jcin.2024.08.016

[9] Grasso C, Baldus S. The MitraClip transcatheter mitral valve repair system. EuroIntervention 2015;14:W45–W46. 10.4244/EIJV11SWA11 26384188

[10] Page MJ, McKenzie JE, Bossuyt PM, Boutron I, Hoffmann TC, Mulrow CD, Shamseer L, Tetzlaff JM, Akl EA, Brennan SE, Chou R, Glanville J, Grimshaw JM, Hróbjartsson A, Lalu MM, Li T, Loder EW, Mayo-Wilson E, McDonald S, McGuinness LA, Stewart LA, Thomas J, Tricco AC, Welch VA, Whiting P, Moher D. The PRISMA 2020 statement: an updated guideline for reporting systematic reviews. BMJ 2021;372:n71. 33782057 10.1136/bmj.n71 PMC8005924

[11] Sterne JA, Hernán MA, Reeves BC, Savović J, Berkman ND, Viswanathan M, Henry D, Altman DG, Ansari MT, Boutron I, Carpenter JR, Chan AW, Churchill R, Deeks JJ, Hróbjartsson A, Kirkham J, Jüni P, Loke YK, Pigott TD, Ramsay CR, Regidor D, Rothstein HR, Sandhu L, Santaguida PL, Schünemann HJ, Shea B, Shrier I, Tugwell P, Turner L, Valentine JC, Waddington H, Waters E, Wells GA, Whiting PF, Higgins JP. ROBINS-I: a tool for assessing risk of bias in non-randomised studies of interventions. BMJ 2016;355:i4919. 27733354 10.1136/bmj.i4919 PMC5062054

[12] Sterne JAC, Savović J, Page MJ, Elbers RG, Blencowe NS, Boutron I, Cates CJ, Cheng HY, Corbett MS, Eldridge SM, Emberson JR, Hernán MA, Hopewell S, Hróbjartsson A, Junqueira DR, Jüni P, Kirkham JJ, Lasserson T, Li T, McAleenan A, Reeves BC, Shepperd S, Shrier I, Stewart LA, Tilling K, White IR, Whiting PF, Higgins JPT. Rob 2: a revised tool for assessing risk of bias in randomised trials. BMJ 2019;366:l4898. 31462531 10.1136/bmj.l4898

[13] Deharo P, Obadia JF, Guerin P, Cuisset T, Avierinos JF, Habib G, Torras O, Bisson A, Vigny P, Etienne CS, Semaan C, Guglieri M, Dumonteil N, Collart F, Gilard M, Modine T, Donal E, Iung B, Fauchier L. Mitral transcatheter edge-to-edge repair vs. isolated mitral surgery for severe mitral regurgitation: a French nationwide study. Eur Heart J 2024;45:940–949. 38243821 10.1093/eurheartj/ehae046

[14] Baldus S, Doenst T, Pfister R, Gummert J, Kessler M, Boekstegers P, Lubos E, Schröder J, Thiele H, Walther T, Kelm M, Hausleiter J, Eitel I, Fischer-Rasokat U, Bufe A, Schmeisser A, Ince H, Lurz P, von Bardeleben RS, Hagl C, Noack T, Reith S, Beucher H, Reichenspurner H, Rottbauer W, Schulze PC, Müller W, Frank J, Hellmich M, Wahlers T, Rudolph V; MATTERHORN investigators. Transcatheter repair versus mitral-valve surgery for secondary mitral regurgitation. N Engl J Med 2024;391:1787–1798. 10.1056/NEJMoa2408739 39216093

[15] Okuno T, Praz F, Kassar M, Biaggi P, Mihalj M, Külling M, Widmer S, Pilgrim T, Grünenfelder J, Kadner A, Corti R, Windecker S, Wenaweser P, Reineke D. Surgical versus transcatheter repair for secondary mitral regurgitation: a propensity score-matched cohorts comparison. J Thorac Cardiovasc Surg 2023;165:2037–2046.e4. 34446288 10.1016/j.jtcvs.2021.07.029

[16] Majmundar M, Patel KN, Doshi R, Kumar A, Arora S, Panaich S, Kalra A. Transcatheter versus surgical mitral valve repair in patients with mitral regurgitation. Eur J Cardiothorac Surg 2024;65:ezad391. 38001034 10.1093/ejcts/ezad391

[17] Koschutnik M, Dannenberg V, Donà C, Nitsche C, Kammerlander AA, Koschatko S, Zimpfer D, Hülsmann M, Aschauer S, Schneider M, Bartko PE, Goliasch G, Hengstenberg C, Mascherbauer J. Transcatheter versus surgical valve repair in patients with severe mitral regurgitation. J Pers Med 2022;12:90. 35055405 10.3390/jpm12010090 PMC8779938

[18] Silaschi M, Cattelaens F, Alirezaei H, Vogelhuber J, Sommer S, Sugiura A, Schulz M, Tanaka T, Sudo M, Zimmer S, Nickenig G, Weber M, Bakhtiary F, Wilde N. Transcatheter edge-to-edge mitral valve repair versus minimally invasive mitral valve surgery: an observational study. J Clin Med 2024;13:1372. 38592259 10.3390/jcm13051372 PMC10932335

[19] Amabile A, Muncan B, Geirsson A, Kalogeropoulos AP, Krane M. Surgical versus interventional mitral valve repair: analysis of 1,100 propensity score-matched patients. J Card Surg 2023;2023:8838005. 10.1111/jocs.16972 36168792

[20] Chikwe J, Chen Q, Bowdish ME, Roach A, Emerson D, Gelijns A, Egorova N. Surgery and transcatheter intervention for degenerative mitral regurgitation in the United States. J Thorac Cardiovasc Surg 2025;169:80–88.e19. 38237762 10.1016/j.jtcvs.2024.01.014

[21] Giustino G, Camaj A, Kapadia SR, Kar S, Abraham WT, Lindenfeld J, Lim DS, Grayburn PA, Cohen DJ, Redfors B, Zhou Z, Pocock SJ, Asch FM, Mack MJ, Stone GW. Hospitalizations and mortality in patients with secondary mitral regurgitation and heart failure: the COAPT trial. J Am Coll Cardiol 2022;80:1857–1868. 36357085 10.1016/j.jacc.2022.08.803

[22] Anker SD, Friede T, von Bardeleben RS, Butler J, Khan MS, Diek M, Heinrich J, Geyer M, Placzek M, Ferrari R, Abraham WT, Alfieri O, Auricchio A, Bayes-Genis A, Cleland JGF, Filippatos G, Gustafsson F, Haverkamp W, Kelm M, Kuck KH, Landmesser U, Maggioni AP, Metra M, Ninios V, Petrie MC, Rassaf T, Ruschitzka F, Schäfer U, Schulze PC, Spargias K, Vahanian A, Zamorano JL, Zeiher A, Karakas M, Koehler F, Lainscak M, Öner A, Mezilis N, Theofilogiannakos EK, Ninios I, Chrissoheris M, Kourkoveli P, Papadopoulos K, Smolka G, Wojakowski W, Reczuch K, Pinto FJ, Wiewiórka Ł, Kalarus Z, Adamo M, Santiago-Vacas E, Ruf TF, Gross M, Tongers J, Hasenfuss G, Schillinger W, Ponikowski P; RESHAPE-HF2 Investigators. Transcatheter valve repair in heart failure with moderate to severe mitral regurgitation. N Engl J Med 2024;391:1799–1809. 39216092 10.1056/NEJMoa2314328

[23] Buzzatti N, Van Hemelrijck M, Denti P, Ruggeri S, Schiavi D, Scarfò IS, Reser D, Taramasso M, Weber A, La Canna G, De Bonis M, Maisano F, Alfieri O. Transcatheter or surgical repair for degenerative mitral regurgitation in elderly patients: a propensity-weighted analysis. J Thorac Cardiovasc Surg 2019;158:86–94.e1. 30797588 10.1016/j.jtcvs.2019.01.023

[24] Grayburn PA, Mack MJ, Manandhar P, Kosinski AS, Sannino A, Smith RL 2nd, Szerlip M, Vemulapalli S. Comparison of transcatheter edge-to-edge mitral valve repair for primary mitral regurgitation outcomes to hospital volumes of surgical mitral valve repair. Circ Cardiovasc Interv 2024;17:e013581. 38436084 10.1161/CIRCINTERVENTIONS.123.013581

[25] Piriou N, Al Habash O, Donal E, Senage T, Le Tourneau T, Pattier S, Guyomarch B, Roussel JC, Trochu JN, Vahanian A, Obadia JF, Iung B, Guérin P. The MITRA-HR study: design and rationale of a randomised study of MitraClip transcatheter mitral valve repair in patients with severe primary mitral regurgitation eligible for high-risk surgery. EuroIntervention 2019;15:e329–e335. 30987963 10.4244/EIJ-D-18-01086

[26] https://clinicaltrials.gov/study/NCT05051033. Last access: 12th August 2025

[27] Suc G, Hadjedj R, Mesnier J, Haviari S, Para M, Ducrocq G, Himbert D, Brochet E, Nguyen ML, Provenchere S, Urena M, Iung B. Transcatheter edge to edge compared with surgery in older patients with degenerative mitral valve regurgitation. J Cardiothorac Surg 2025;20:65. 39815350 10.1186/s13019-024-03257-x PMC11736988

