# Supplementary material for: Mitral Transcatheter edge-to-edge repair rivals surgery for survival despite less complete correction: a systematic review and metanalysis of randomized and propensity score matching studies
Source: Eur Heart J Open. 2025 Oct 25;5(6):oeaf135. doi: 10.1093/ehjopen/oeaf135 (PMC12604465; doi:10.1093/ehjopen/oeaf135)

***Appendix S1 – Included studies***

1. Deharo P, Obadia JF, Guerin P, Cuisset T, Avierinos JF, Habib G, Torras O, Bisson A, Vigny P, Etienne CS, Semaan C, Guglieri M, Dumonteil N, Collart F, Gilard M, Modine T, Donal E, Iung B, Fauchier L. Mitral transcatheter edge-to-edge repair vs. isolated mitral surgery for severe mitral regurgitation: a French nationwide study. Eur Heart J. 2024 Mar 14;45(11):940-949. doi: 10.1093/eurheartj/ehae046. PMID: 38243821.
2. Baldus S, Doenst T, Pfister R, Gummert J, Kessler M, Boekstegers P, Lubos E, Schröder J, Thiele H, Walther T, Kelm M, Hausleiter J, Eitel I, Fischer-Rasokat U, Bufe A, Schmeisser A, Ince H, Lurz P, von Bardeleben RS, Hagl C, Noack T, Reith S, Beucher H, Reichenspurner H, Rottbauer W, Schulze PC, Müller W, Frank J, Hellmich M, Wahlers T, Rudolph V; MATTERHORN Investigators. Transcatheter Repair versus Mitral-Valve Surgery for Secondary Mitral Regurgitation. N Engl J Med. 2024 Nov 14;391(19):1787-1798. doi: 10.1056/NEJMoa2408739. Epub 2024 Aug 31. PMID: 39216093.
3. Okuno T, Praz F, Kassar M, Biaggi P, Mihalj M, Külling M, Widmer S, Pilgrim T, Grünenfelder J, Kadner A, Corti R, Windecker S, Wenaweser P, Reineke D. Surgical versus transcatheter repair for secondary mitral regurgitation: A propensity score-matched cohorts comparison. J Thorac Cardiovasc Surg. 2023 Jun;165(6):2037-2046.e4. doi: 10.1016/j.jtcvs.2021.07.029. Epub 2021 Jul 28. PMID: 34446288.
4. Majmundar M, Patel KN, Doshi R, Kumar A, Arora S, Panaich S, Kalra A. Transcatheter versus surgical mitral valve repair in patients with mitral regurgitation. Eur J Cardiothorac Surg. 2024 Jan 2;65(1):ezad391. doi: 10.1093/ejcts/ezad391. PMID: 38001034 (surgical mitral valve repair)
5. Koschutnik M, Dannenberg V, Donà C, Nitsche C, Kammerlander AA, Koschatko S, Zimpfer D, Hülsmann M, Aschauer S, Schneider M, Bartko PE, Goliasch G, Hengstenberg C, Mascherbauer J. Transcatheter Versus Surgical Valve Repair in Patients with Severe Mitral Regurgitation. J Pers Med. 2022 Jan 11;12(1):90. doi: 10.3390/jpm12010090. PMID: 35055405; PMCID: PMC8779938.
6. Feldman T, Kar S, Elmariah S, Smart SC, Trento A, Siegel RJ, Apruzzese P, Fail P, Rinaldi MJ, Smalling RW, Hermiller JB, Heimansohn D, Gray WA, Grayburn PA, Mack MJ, Lim DS, Ailawadi G, Herrmann HC, Acker MA, Silvestry FE, Foster E, Wang A, Glower DD, Mauri L; EVEREST II Investigators. Randomized Comparison of Percutaneous Repair and Surgery for Mitral Regurgitation: 5-Year Results of EVEREST II. J Am Coll Cardiol. 2015 Dec 29;66(25):2844-2854. doi: 10.1016/j.jacc.2015.10.018. PMID: 26718672
7. Silaschi M, Cattelaens F, Alirezaei H, Vogelhuber J, Sommer S, Sugiura A, Schulz M, Tanaka T, Sudo M, Zimmer S, Nickenig G, Weber M, Bakhtiary F, Wilde N. Transcatheter Edge-to-Edge Mitral Valve Repair versus Minimally Invasive Mitral Valve Surgery: An Observational Study. J Clin Med. 2024 Feb 28;13(5):1372. doi: 10.3390/jcm13051372. PMID: 38592259; PMCID: PMC10932335
8. Amabile, Andrea, Muncan, Brandon, Geirsson, Arnar, Kalogeropoulos, Andreas P., Krane, Markus, Surgical versus Interventional Mitral Valve Repair: Analysis of 1,100 Propensity Score-Matched Patients, *Journal of Cardiac Surgery*,  2023, 8838005, 7 pages,  2023. <https://doi.org/10.1155/2023/8838005>
9. Chikwe J, Chen Q, Bowdish ME, Roach A, Emerson D, Gelijns A, Egorova N. Surgery and transcatheter intervention for degenerative mitral regurgitation in the United States. J Thorac Cardiovasc Surg. 2025 Jan;169(1):80-88.e19. doi: 10.1016/j.jtcvs.2024.01.014. Epub 2024 Jan 17. PMID: 38237762.

***Appendix S2 – Excluded studies***

1. Buzzatti N, Van Hemelrijck M, Denti P, Ruggeri S, Schiavi D, Scarfò IS, Reser D, Taramasso M, Weber A, La Canna G, De Bonis M, Maisano F, Alfieri O. Transcatheter or surgical repair for degenerative mitral regurgitation in elderly patients: A propensity-weighted analysis. J Thorac Cardiovasc Surg. 2019 Jul;158(1):86-94.e1
2. Ahmed A, Eisa M, Takla A, Arora S, Mohamed MS, Hanafi A, Feitell S. Temporal trends and procedural safety of mitral valve transcatheter edge to edge repair in patients with previous CABG. Future Cardiol. 2025 Jan;21(1):15-22.

**Figure S1 – Violin plots showing the approximate distribution of key baseline characteristics in m-TEER and surgical cohorts**


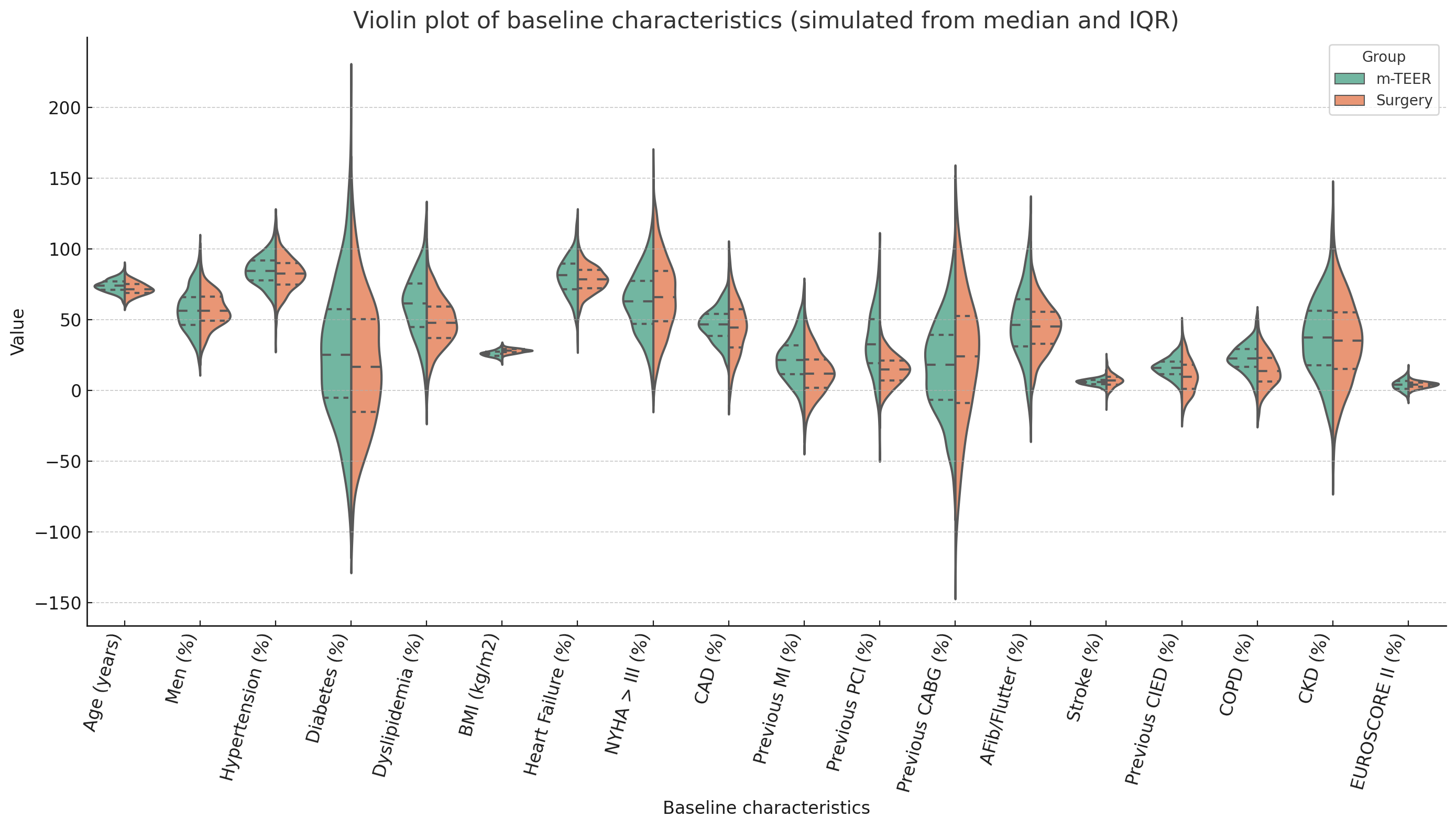


**Figure S2 – Risk of bias assessment**

Randomized controlled trials – ROB2 tool


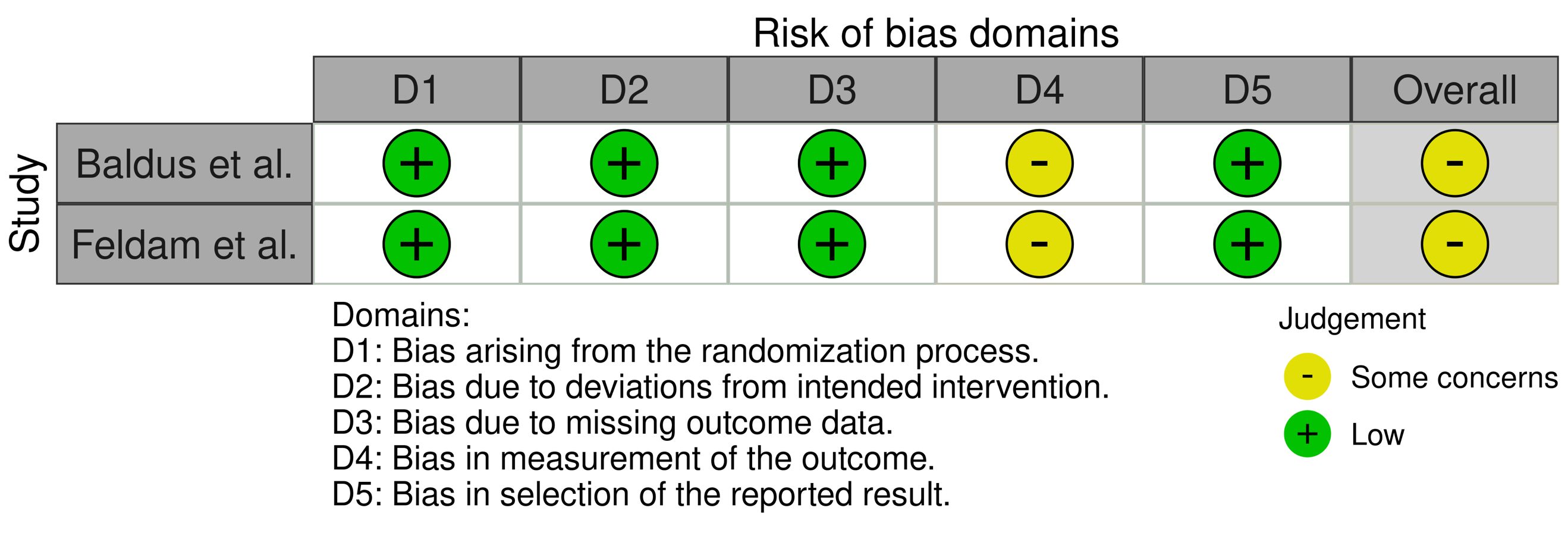


Propensity score matching studies– ROBINS I tool


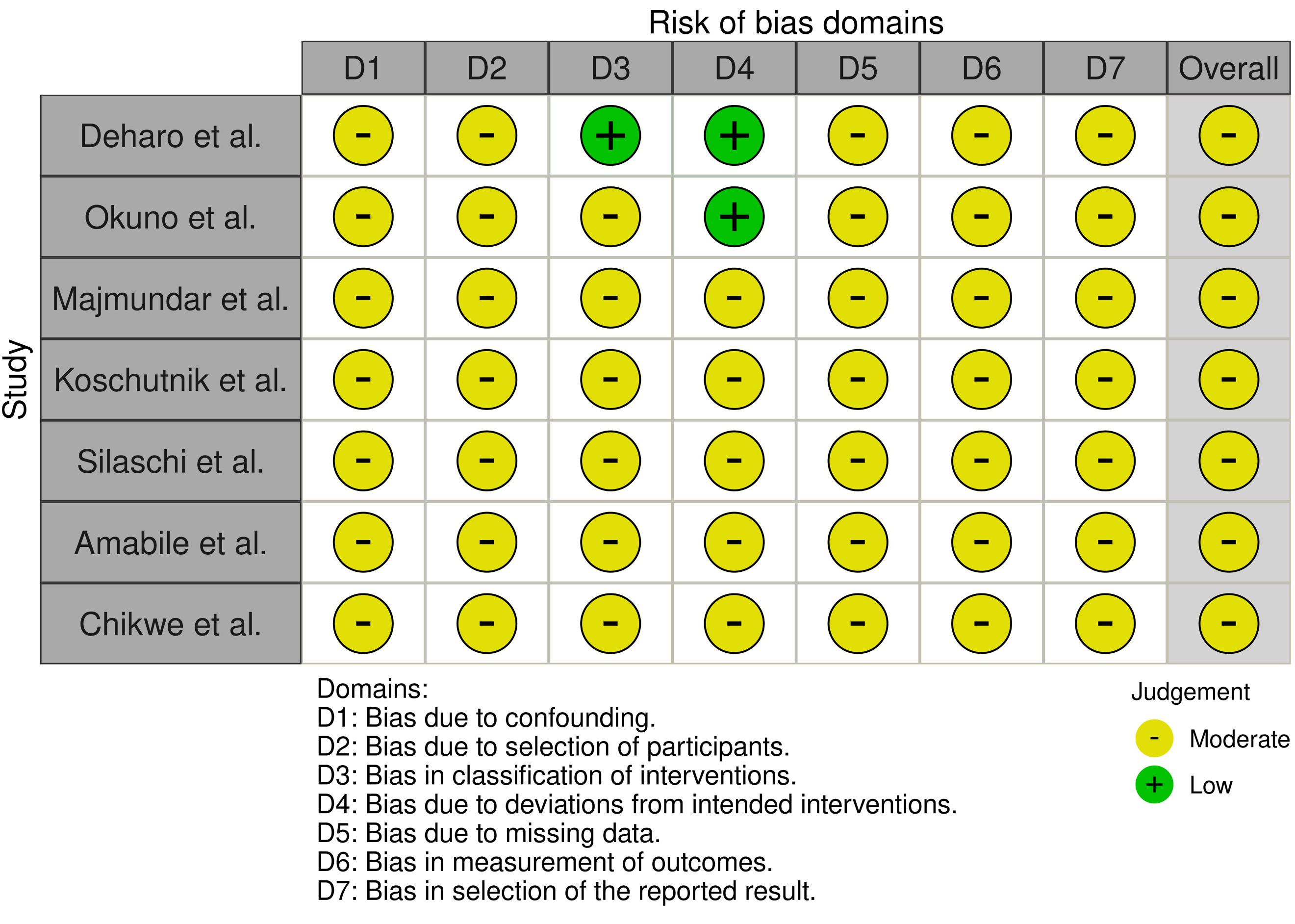


Figure S3 - Funnel Plot All-cause of death


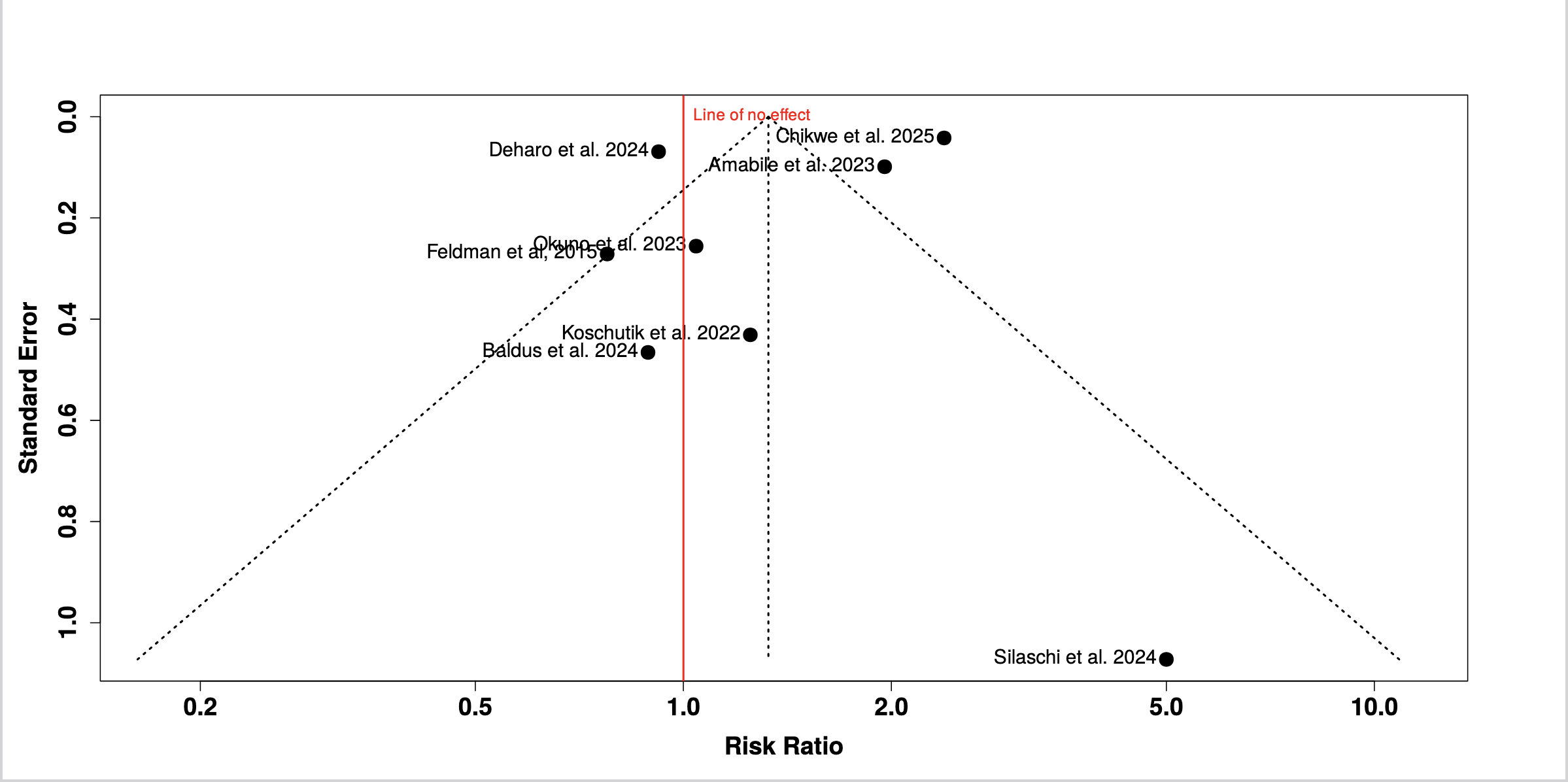


**Figure S4 – Mitral Reintervention – Subgroup analysis m-TEER versus MV repair**


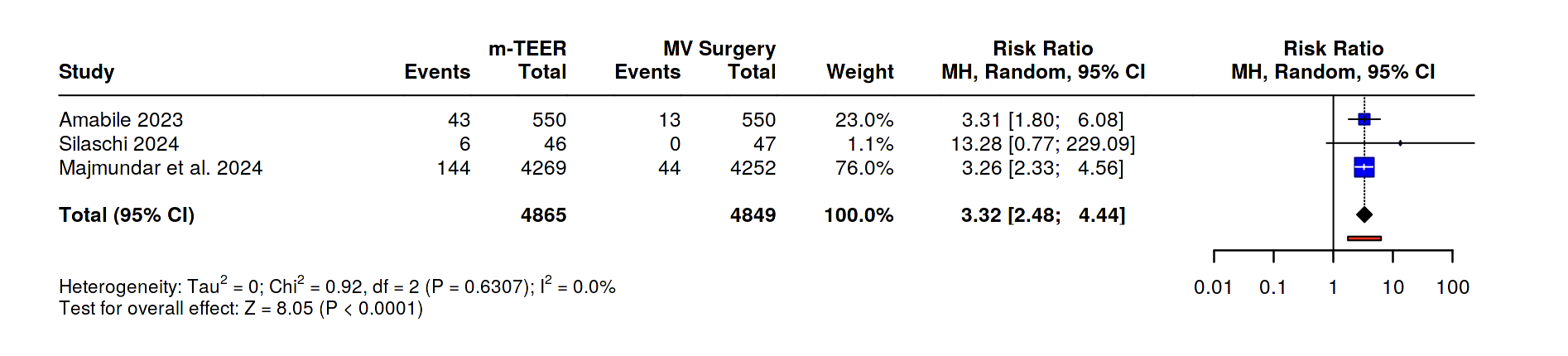


Figure S5 - Grade ≥ 3 at echocardiographic follow-up. Forrest Plot (A). Visual Bar Plot (B) using the Clopper-Pearson method

A


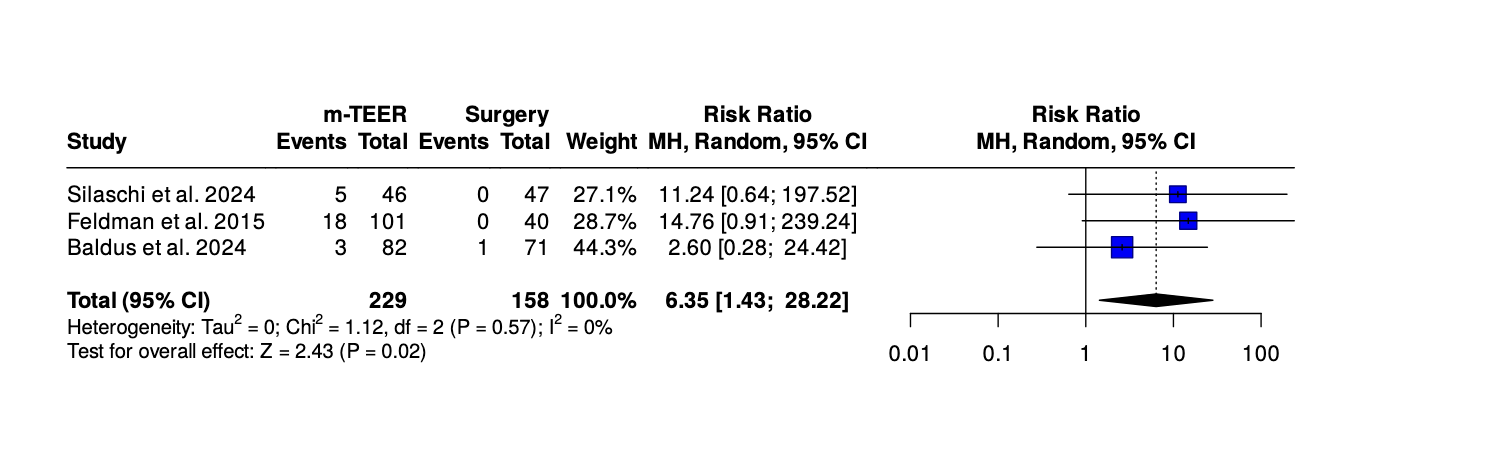


B


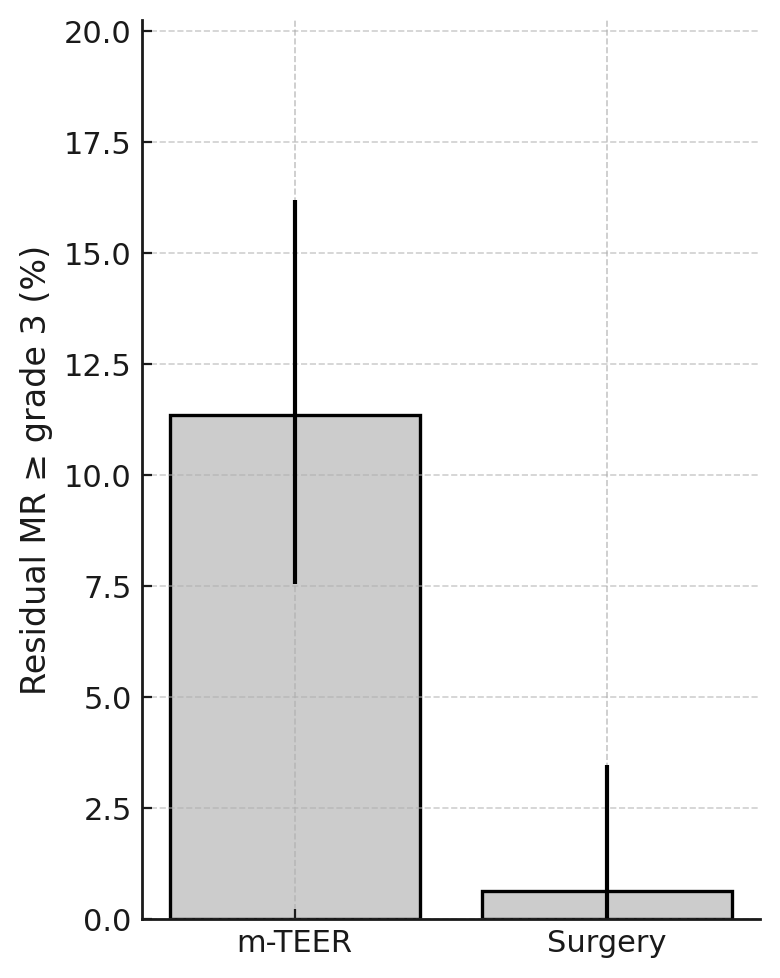


Figure S6 – NYHA Class ≥ 3 at clinical follow-up


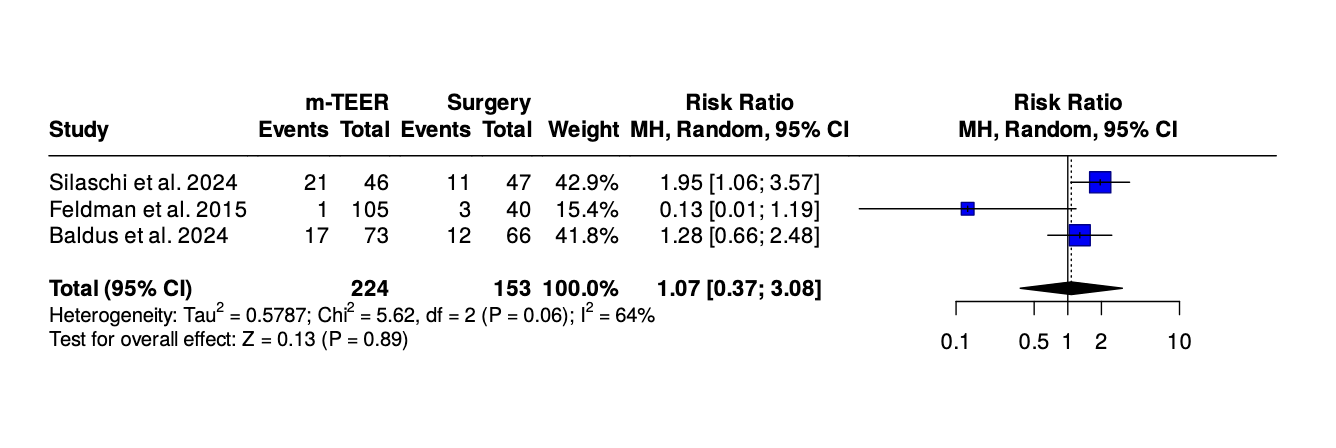


Figure S7- All cause of death without EVEREST II Trial. Primary (A) and sensitive analysis (B).


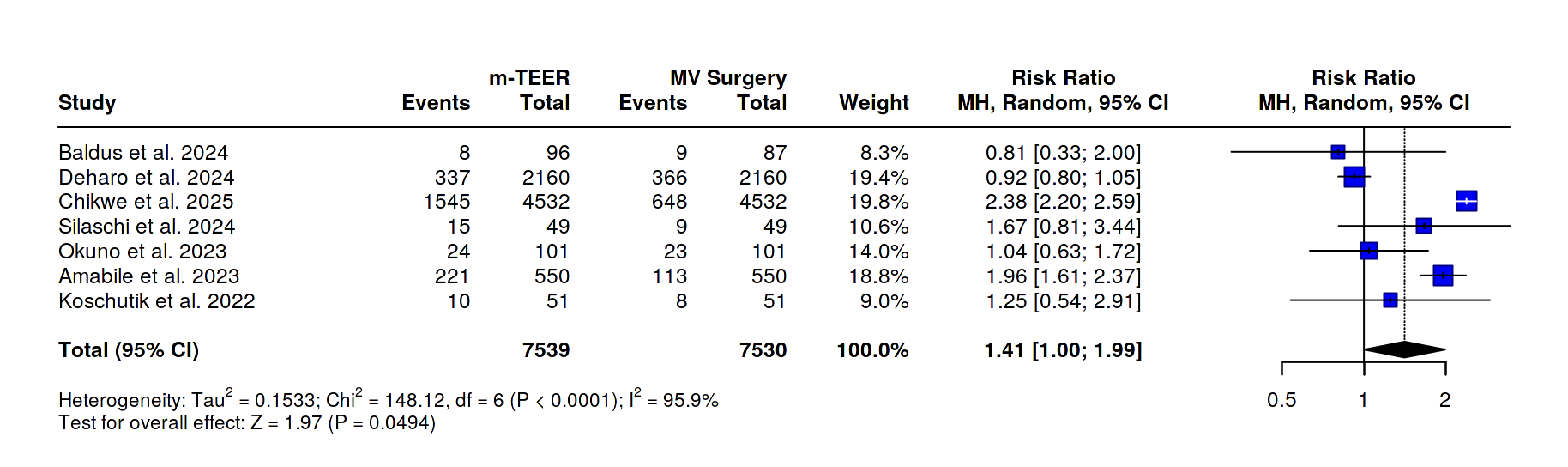


A


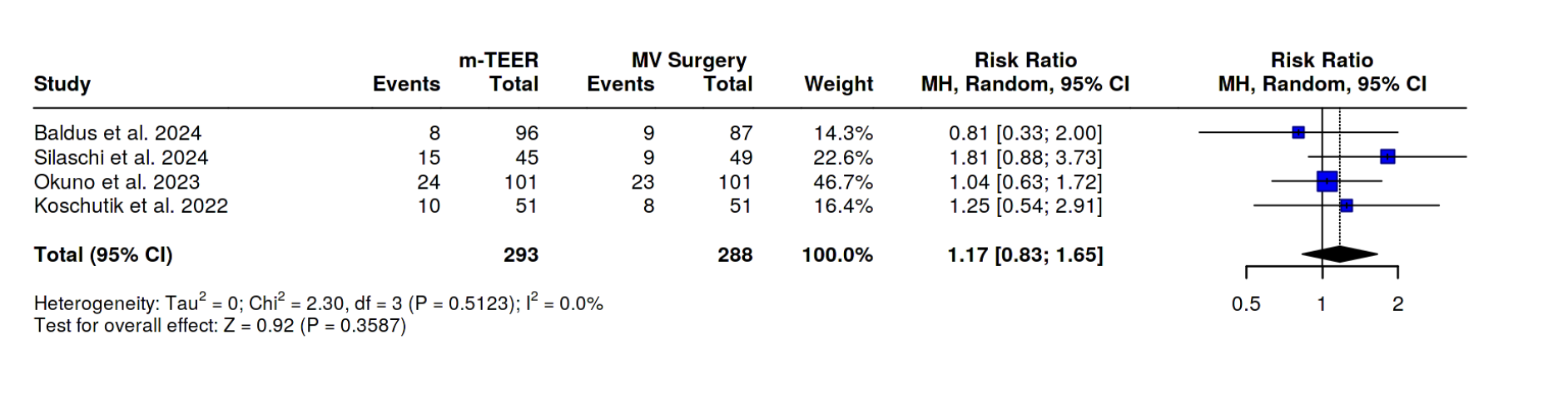


B

Figure S8 – Mitral Reintervention without EVEREST II trial. Primary (A) and sensitive analysis (B).


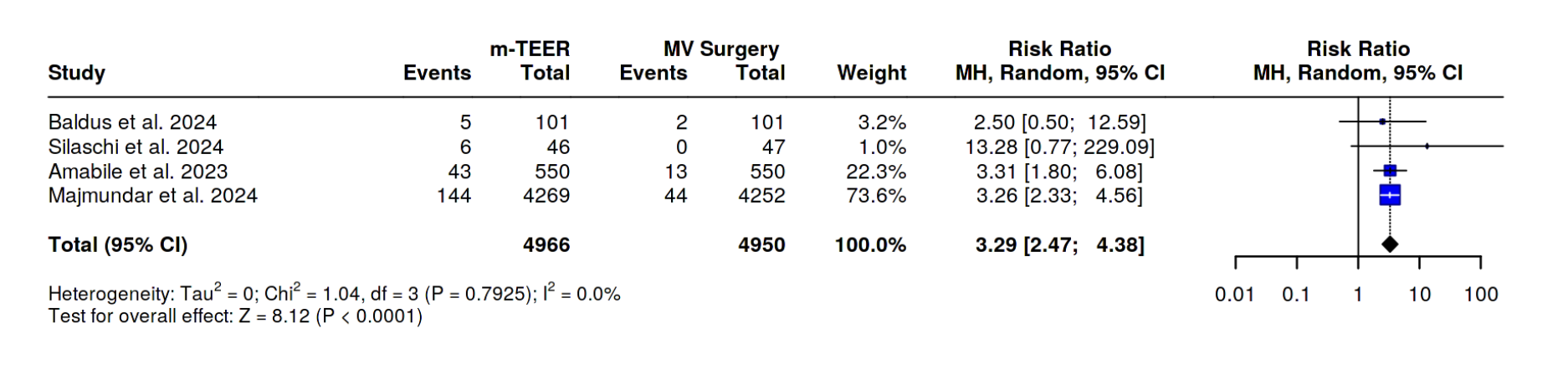

Supplement: oeaf135_Supplementary_Data [file oeaf135_supplementary_data.docx]
